# Psychiatric and forensic aspects of homicide-suicide and prevention perspectives in offenders: a review of the literature

**DOI:** 10.1192/j.eurpsy.2023.1876

**Published:** 2023-07-19

**Authors:** M. A. Sacco, F. Cordasco, C. Scalise, M. Maesani, S. Gualtieri, A. P. Tarallo, P. Ricci, I. Aquila

**Affiliations:** 1 Institute of Legal Medicine University Magna Graecia of Catanzaro, Catanzaro, Italy

## Abstract

**Introduction:**

Homicide-suicide is a rarer phenomenon than homicides or suicides alone. However, this event shows a devastating psychological impact on relatives with effects on the mental health of survivors. The possibility of early intervention through the identification of psychopathological risk factors in offenders remains a challenge of current forensic psychiatry, also for the geographic heterogeneity of the phenomenon.

**Objectives:**

The purpose of the work is to offer a homogeneous review of the state of the art on the homicide-suicide phenomenon, focusing attention on the psychopathological characteristics of the offenders and related prevention.

**Methods:**

A literature review was carried out through the PubMed NCBI search engine by entering the keywords *“homicide-suicide” and psychiatry.* A screening of the titles was carried out, with reading of the abstracts and full texts only in the selected papers that respected the research objectives.

**Results:**

The search evidenced 71 papers and only 20 met the inclusion criteria. The review revealed in the vast majority of offenders, most often male, the presence of mental disorders such as major depression and schizophrenia, especially in the context of relationship problems, with triggers such as the fear of separation or partner unfaithfulness. In these cases, the relationship between victim and killer shows pathological dependence or extreme possession. The event can involve children or relatives (filicide/familicide suicides). The review showed the frequent association with psychiatric depressive disorders, and obsessive-compulsive, paranoid, or narcissistic traits, with a lack of specialist consultations and very poor adherence to psychiatric treatment. Firearms were the most widely used means. In the history of perpetrators there were frequent episodes of domestic violence, previous suicide attempts and substance abuse. In rarer cases, the event takes place in the context of mass murder-suicides in which the offender assumes positions of responsibility (eg. Airplane pilot) or acts in extremist religious contexts.

**Conclusions:**

The review showed an association with mental health disorders and the need for early identification of risk signals. Appropriate psychiatric prevention programs, through the close collaboration of the family, social community and the mass media, are necessary in the management of the phenomenon, especially through the reporting of episodes of domestic violence or self-harming attempts.

**Disclosure of Interest:**

None Declared

